# *Mycobacterium senegalense* Osteomyelitis of the Distal Tibia: A Case Report

**DOI:** 10.7150/jbji.33321

**Published:** 2019-05-21

**Authors:** Jeremiah Maupin, Austin Cantrell, Katherine Kupiec, Dante Paolo Melendez, Amgad M. Haleem

**Affiliations:** 1 Department of Orthopedic Surgery and Rehabilitation, University of Oklahoma Health Sciences Center, Oklahoma City, Oklahoma, USA; 2 Department of Pharmacy, University of Oklahoma Medical Center, Oklahoma City, Oklahoma, USA; 3 Department of Internal Medicine, Infectious Disease Section, University of Oklahoma Health Sciences Center, Oklahoma City, Oklahoma, USA; 4 Department of Orthopedic Surgery, College of Medicine, Cairo University, Cairo, Egypt

**Keywords:** Mycobacterium senegalense, osteomyelitis, Nontuberculous mycobacterium, Ilizarov External Fixator

## Abstract

*Mycobacterium senegalense* infection is rare. We present the third documented case of *M. senegalense* infection and the first to involve the musculoskeletal system. A 55-year old immunocompetent male developed chronic osteomyelitis of the ankle and required antibiotic spacers, an Ilizarov external fixator and multiple antibiotic regimens to eradicate the infection.

## Introduction

*Mycobacterium senegalense* is a nontuberculous mycobacterium (NTM) species in the *Mycobacterium fortuitum* group best known for its devastating effects on cattle as one of the two leading causal agents of bovine farcy along with *Mycobacterium farcinogenes*.^1^ Characterized by multiple abscesses, draining sinus tracts, and granuloma formation, bovine farcy leads to emaciation, death, and ultimately the inability to harvest beef from afflicted cattle causing both a disease and economic burden to ranchers.

While NTM infections occur in humans, it is difficult to predict an accurate incidence because unlike *Mycobacterium tuberculosis*, there is no mandatory health reporting for NTM infections.^2^ NTM infections can affect multiple organ systems including the pulmonary, integumentary, hematologic and musculoskeletal systems.^3^ More than 120 different species of NTM can cause human infection^2^ and there is considerable difficulty in treating these infections. Many NTM species are resistant to routine anti-tuberculosis drugs, 4, 5 and with the slow growth rate of mycobacteria, patients often require prolonged durations of combination therapy which can lead to various side effects and difficulties tolerating treatment.

While human infection with *M. senegalense* appears to be rare with only two cases reported in the literature.^6^,^7^, there is likely an underestimation of the true incidence of these cases as some laboratories do not subspeciate *M. fortuitum* complex infections. The first reported case of *M. senegalense* occurred as a bacteremia from a central line infection in an immunocompromised 49 year-old female.^7^ The second reported case occurred as a soft-tissue infection in a 3 year-old female who sustained facial lacerations from the glass of a freshwater fish tank.^6^ Here, we present the third reported case of *M. senegalense* infection and the first to demonstrate musculoskeletal involvement in a 55 year-old male who developed chronic osteomyelitis following a traumatic open ankle fracture.

## Methods

A literature search of the PubMed database was performed without time restrictions to locate reports of *M. senegalense* infection with the search term “senegalense.” 65 total reports were identified spanning 1978-2018 and each was assessed for applicability to human infection. A total of 2 reports documented human infections and both were reviewed and referenced in this manuscript.

### Case Report

A 55-year-old immunocompetent male was involved in a motor vehicle collision 2 years prior to presenting to our institution. He sustained an open ankle fracture after being ejected from the driver's seat into a pasture ditch, sustaining lacerations from barbed wire and resulting in a grossly contaminated open fracture. He was initially managed with irrigation and debridement (I&D) and placement of an external fixator. He subsequently underwent definitive fixation but during the next two years, experienced episodic drainage from incisions that was managed symptomatically with oral antibiotics. He had persistent pain which led him to seek evaluation at our institution.

**Figure 1:**
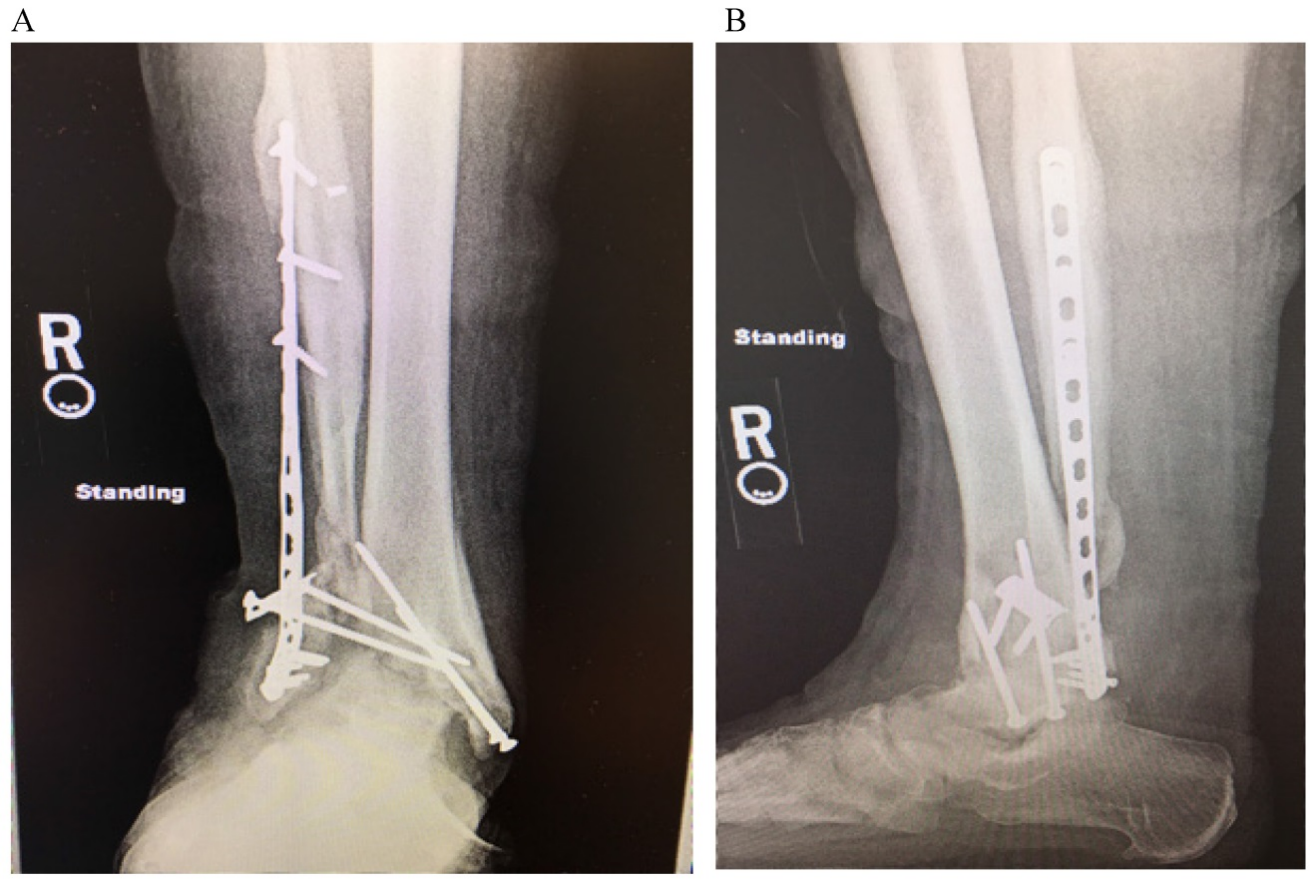
AP (A) and lateral (B) radiograph demonstrating hardware failure and destruction of the tibiotalar joint with severe valgus deformity and non-union

**Figure 2:**
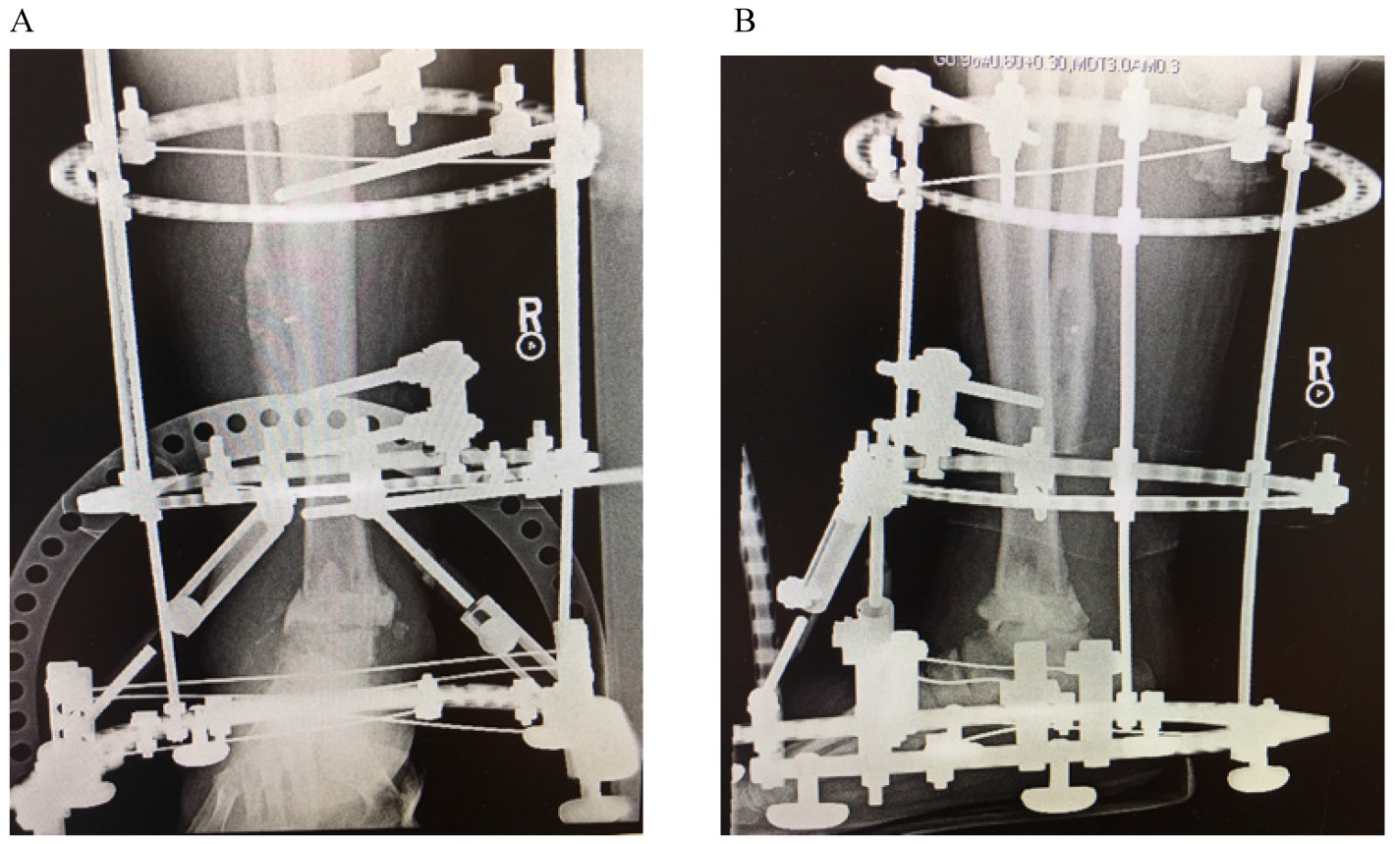
AP (A) and lateral (B) radiographs following removal of hardware, placement of tibiotalar antibiotic spacer and ringed external fixator

Upon presentation to our clinic, he was noted to have a severe planovalgus alignment, two draining sinuses, radiographic hardware failure (Figure 1), and elevated inflammatory markers including a white blood cell (WBC) count of 11 K/mm^3^, C-reactive protein (CRP) of 18.7 mg/L, and Erythrocyte Sedimentation Rate (ESR) of 18 mm/hr. He was diagnosed with an infected nonunion with underlying chronic septic arthritis of the tibiotalar joint and osteomyelitis of the distal tibia and fibula and scheduled for a staged ankle fusion following eradication of his infection. At our index surgery, the patient underwent a complete hardware removal with the exception of a broken screw tip from the most proximal fibular screw, extensive debridement of the ankle including excision of 11 centimeters (cm) of diseased distal fibula, 1.5cm of tibial plafond, and 0.5cm of talar dome, placement of an antibiotic spacer to the residual tibiotalar joint (impregnated with 2 grams of vancomycin and 2.4 grams of tobramycin per 40 grams of cement), and application of a ringed (Ilizarov) external fixator (Figure 2). Intraoperatively, multiple tissue cultures were obtained and found to be positive for *Enterococcus faecalis*, *Enterobacter cloacae*, and *Mycobacterium senegalense,* which grew from regular bacterial cultures.

Postoperatively, the patient was started on a 4-week regimen of piperacillin-tazobactam for the *E. faecalis* and *E. cloacae* osteomyelitis followed by a 6-week regimen of imipenem, ciprofloxacin, and minocycline for his *M. senegalense* based on the *in vitro* antibiotic susceptibilities from the culture (Table 1). Three months after his index surgery, he continued to have drainage from 2 sites and the decision was made to undergo a repeat I&D with placement of a new antibiotic spacer (impregnated with vancomycin and tobramycin). Intraoperatively, the distal tibia had the appearance of persistent infection including purulence and caseous material which was cultured and noted to be positive for *Bacillus* species (not anthracis) and persistent *M. senegalense* growth, again from regular bacterial cultures (Table 1). At this point, based on the recurrent isolation of *M. senegalense*, the decision was made to continue imipenem and change the oral antibiotics to linezolid and azithromycin for another three months. Finally, after finishing 3 more months of imipenem, it was replaced with doxycycline to complete another 3 months of an all oral regimen.

**Figure 3:**
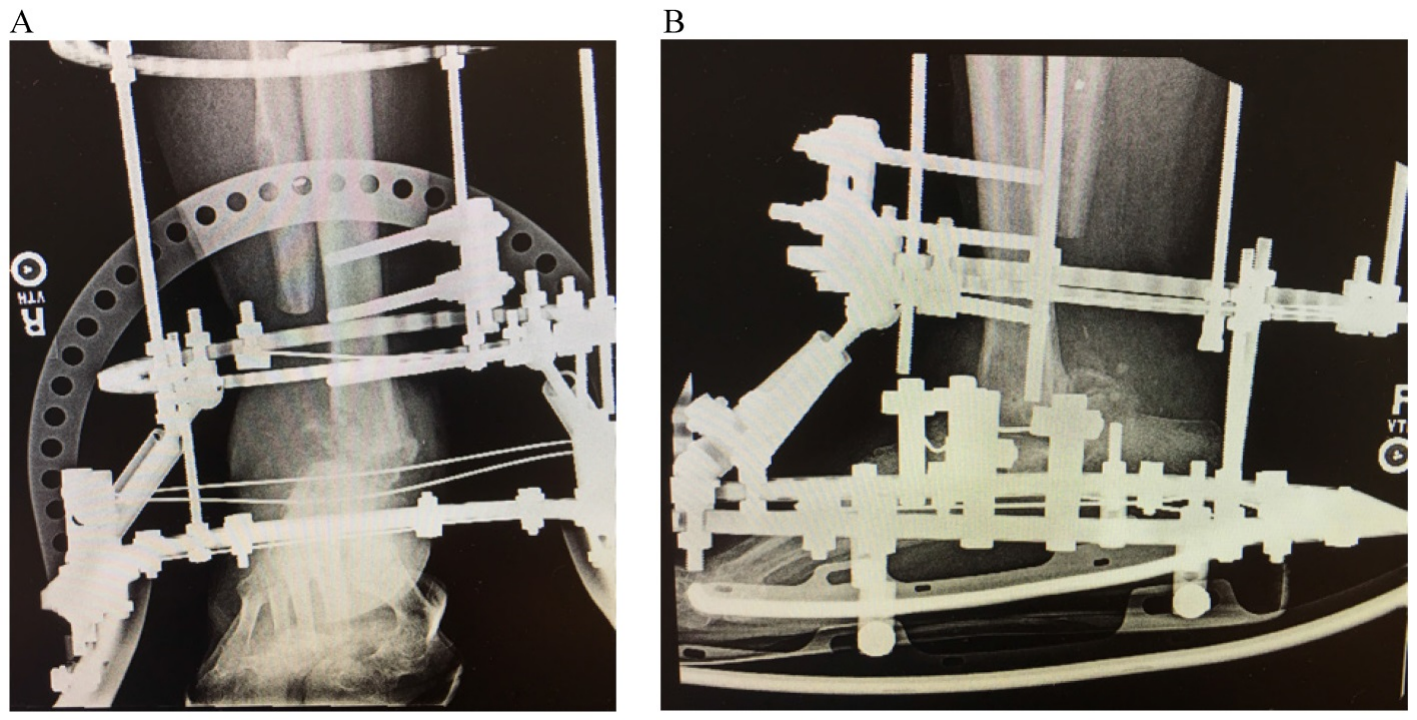
AP (A) and lateral (B) radiographs following definitive ankle fusion after eradication of infection

Four months later, he had closure of all wounds, demonstrated downtrending inflammatory markers (WBC, ESR, CRP), and was felt to be ready for fusion. During the procedure, no purulence was encountered, iliac crest autograft was utilized to augment the tibiotalar fusion and compression through the joint was achieved through the ringed external fixator (Figure 3). Multiple tissue cultures were taken intraoperatively with no growth noted and postoperatively he was continued on his regimen of linezolid, azithromycin, and doxycycline.

**Figure 4:**
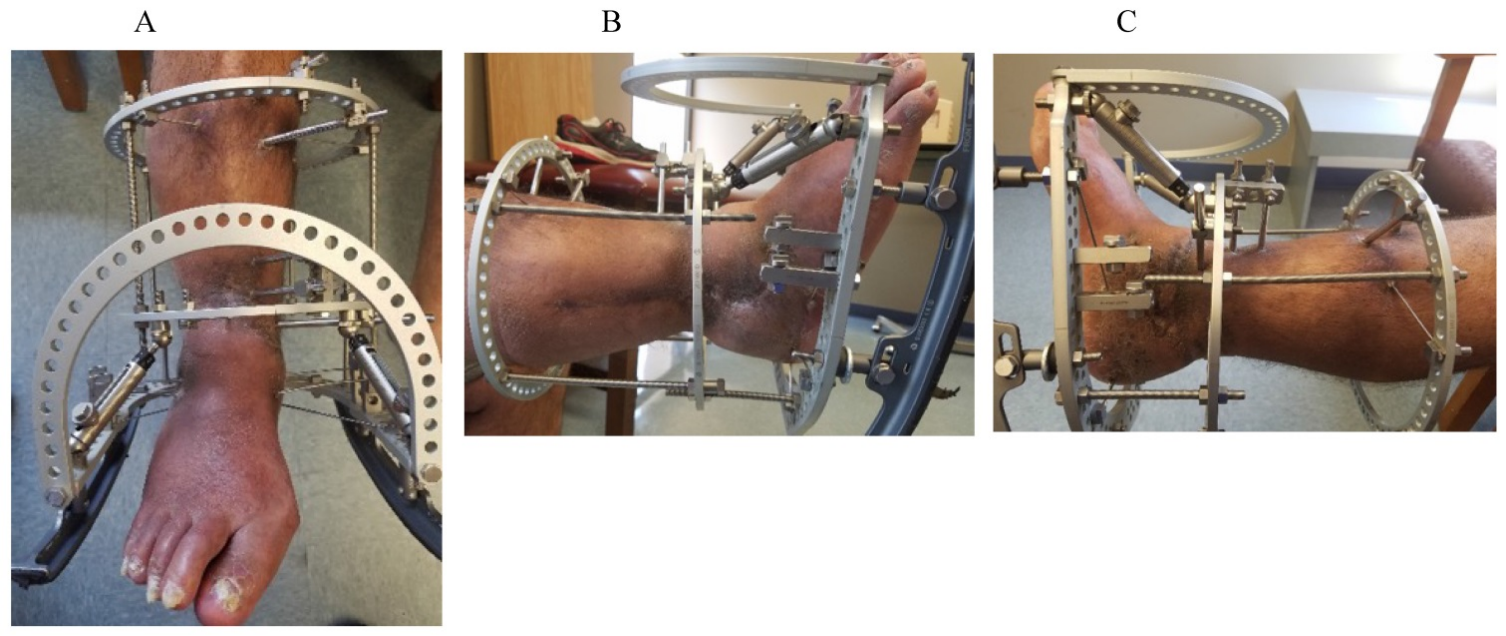
Right lower extremity (A-C) following successful infection eradication and tibiotalar fusion demonstrating healing of prior surgical sites and sinus tracts.

**Figure 5:**
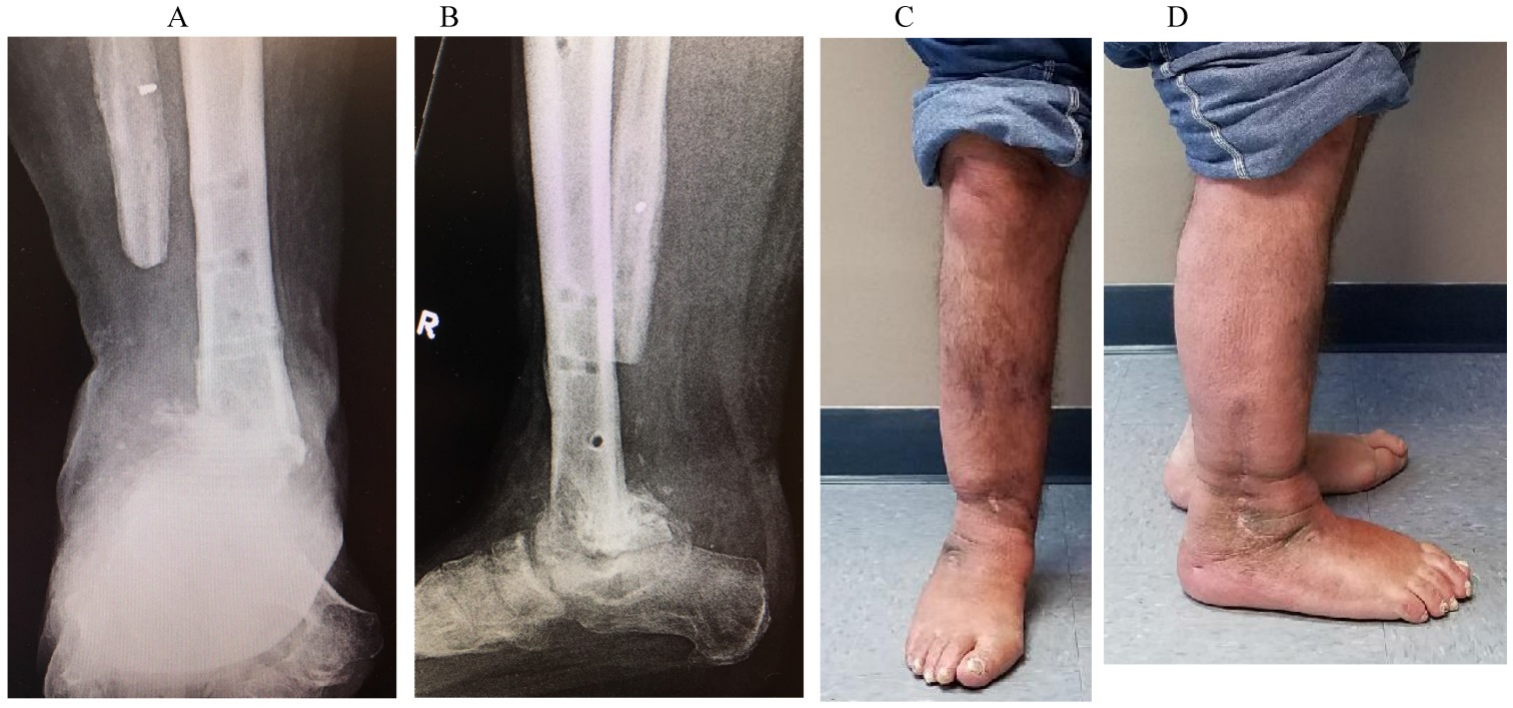
AP (A) and Lateral (B) radiographs with same day clinical appearance of extremity (C & D) 1-year following fusion demonstrating a solid tibiotalar fusion mass and well healed incisions with no evidence of infection recurrence.

Six months postoperatively, he had a successful fusion with no sinus tracts or drainage (Figure 4). He was able to have his ringed external fixator removed and one year postoperatively the patient continues to do well, fully weightbearing through the extremity and has not shown any signs of recurrence (Figure 5).

## Discussion

Originally thought to be saprophytic bacteria, it was not until the 1950's that NTM species were recognized as human pathogens.^8^ Now, there have been more than 120 different species of NTM identified that are capable of causing human disease.^2^ The increased incidence of NTM infections is thought to be partially attributable to the genesis of the Acquired Immunodeficiency Syndrome (AIDS) epidemic, the use of immunosuppressive therapies, and the more widespread use of mycobacterial cultures and molecular identification methods in the laboratory.^9^ The significant increase in the incidence of infections over the past 60 years, the associated disease burden that comes with management of these infections, and the increased resistance of NTM infections to routine antituberculosis drugs has led NTM species to gain acknowledgement as important pathogens of disease 4,5.

This case demonstrates how difficult NTM infections can be to treat and the importance of a multidisciplinary team in management. From initial presentation to our facility, it was nearly a full year of both surgical and medical management before the infection was eradicated. While NTM species have the potential to be a contaminant, we maintain confidence that *M. senegalense* was causing infection in our case as it was isolated on two separate occasions 3 months apart.

As literature on *M. senegalense* infection is sparse and limited to two prior case reports, we based our antimicrobial management on the available guidelines for NTM infection treatment on *M. fortuitum* group, to which *M. senegalense* belongs.^10^ Thus, we treated with a combination of a minimum of 2 antimicrobials specifically directed to this isolate of *M. senegalense*, guided by *in vitro* susceptibilities.

While macrolides are the backbone in the treatment of most NTM infections, current treatment guidelines by the American Thoracic Society/Infectious Diseases Society of America recommend caution in their use when treating infections by the *M. fortuitum* group, given the potential for development of resistance through an inducible erythromycin methylase *erm* gene.^10^ Testing for this *erm* gene-related resistance requires incubation of the mycobacterial isolate in the presence of a macrolide for 14 days before the assessment of its minimum inhibitory concentration. This technique is unfortunately not widely available and as such, was not available in this case. While the initial antibiotic regimen consisted of 3 non-macrolide agents, the definitive antibiotic regimen included a macrolide in addition to other 2 antibiotics, one of which was administered intravenously. We cautiously used the macrolide antibiotic only as an addition to the other 2 non-macrolide antibiotics and after extensive surgical debridement had been performed. This multimodal approach of thorough surgical debridement with removal of infected hardware and combination antibiotic therapy is key in the treatment of NTM infections and in our opinion decreased substantially the risk for development of macrolide resistance and increased the chances of success. To further illustrate this point, the previously reported *M senegalense* catheter-related infection failed initial conservative management but was successfully treated only after fully removing the infected hardware 7.

From a surgical standpoint, chronic osteomyelitis of long bones can be a serious and frustrating problem to treat as there is no generally accepted consensus on management. At our institution, we typically manage cases of chronic osteomyelitis in a staged fashion, allowing us to confirm eradication prior to definitive surgery. For cases like this with significant periarticular joint destruction, we favor the use of an Ilizarov external fixator which allows us the versatility to maintain the hardware away from the infected tissues while simultaneously allowing us access to the site of infection for serial debridements, such as in this case where an antibiotic spacer exchange was necessary. Furthermore, the Ilizarov external fixator has the added benefit of being capable of generating compression across the fusion sites at the time of definitive surgery, as well as post-operatively in an outpatient setting if needed. Prior studies have shown success managing chronic osteomyelitis with Ilizarov frames when combined with appropriate antibiotic therapy. While there has not been literature specifically addressing NTM infections managed with Ilizarov external fixators, eradication was achieved in our case and we anticipate success rates would be similar to those of prior published studies 11,12.

## Conclusion

Initially thought to be benign saprophytes, nontuberculous mycobacterium species are now a well-known source of human infection. This case demonstrates the third documented case of human *Mycobacterium senegalense* infection and is the first to be associated with osteomyelitis. Through a multidisciplinary approach including infectious disease specialists and orthopedic surgeons, we were ultimately able to eradicate the infection and preserve the patient's extremity. We present this case to further add to the sparse body of literature on this species and create awareness that *M. senegalense* can be a source of osteomyelitis, even in an immunocompetent host.

## Author contributions

AMH and DPM are the attending physicians for the patient and collected medical data for the patient. JM is the resident physician for the patient. KK is the pharmacist consulting on the case. JM, AC, KK, DPM, and AMH all contributed to the manuscript. All authors have read and approved the final manuscript.

## Figures and Tables

**Table 1:** Culture sensitivities from *Mycobacterium senegalense* isolates

Antibiotic	Index Culture MIC (mcg/mL)	Interpretation	Second Culture MIC (mcg/mL)	Interpretation
Amikacin	<=8	S	<=8	S
Amoxicillin-Clavulanate	8/4	S	16/8	I
Azithromycin	<=16	S	<=16	S
Cefepime	>32	R	>32	R
Cefotaxime	>64	R	>64	R
Cefoxitin	32	I	32	R
Ciprofloxacin	<=1	S	<=1	S
Clarithromycin	<=0.25	S	<=0.25	S
Clofazimine	<=0.5	S	<=0.5	S
Ceftriaxone	>64	R	>64	R
Doxycycline	<=1	S	<=1	S
Gentamicin	<=2	S	<=2	S
Imipenem	<=2	S	<=2	S
Kanamycin	<=8	S	<=8	S
Linezolid	4	S	4	S
Minocycline	<=1	S	<=1	S
Moxifloxacin	<=0.5	S	<=0.5	S
Tigecycline	<=0.25	S	0.25	S
Tobramycin	4	S	4	S
Trimethoprim-Sulfamethoxazole	4/76	R	1/19	S
*MIC-Minimum Inhibitory Concentration; S-Sensitive; R-Resistant; I-Indeterminate				

## Abbreviations

NTM: nontuberculous mycobacterium
I&D: irrigation and debridement
WBC: white blood cell
CRP: C-reactive protein
ESR: Erythrocyte Sedimentation Rate
AIDS: Acquired Immunodeficiency Syndrome
MIC: minimum inhibitory concentration
